# Characterisation of Hyaluronic Acid Blends Modified by Poly(*N*-Vinylpyrrolidone)

**DOI:** 10.3390/molecules26175233

**Published:** 2021-08-29

**Authors:** Katarzyna Lewandowska, Marta Szulc

**Affiliations:** Faculty of Chemistry, Nicolaus Copernicus University in Toruń, Gagarin 7, 87-100 Toruń, Poland; marta.sz@doktorant.umk.pl

**Keywords:** polymer blends, rheological properties, poly(*N*-vinylpyrrolidone), hyaluronic acid

## Abstract

The viscosity behaviour and physical properties of blends containing hyaluronic acid (HA) and poly(*N*-vinylpyrrolidone) (PVP) were studied by the viscometric technique, steady shear tests, tensile tests and infrared spectroscopy. Viscometric and rheological measurements were carried out using blends of HA/PVP with different HA weight fractions (0, 0.2, 0.5, 0.8 and 1). The polymer films and HA/PVP blend films were prepared using the solution casting method. The study of HA blends by viscometry showed that HA/PVP was miscible with the exception of the blend with high HA content. HA and its blends showed a shear-thinning flow behaviour. The non-Newtonian indices (*n*) of HA/PVP blends were calculated by the Ostwald–de Waele equation, indicating a shear-thinning effect in which pseudoplasticity increased with increasing HA contents. Mechanical properties, such as tensile strength and elongation at the break, were higher for HA/PVP films with w_HA_ = 0.5 compared to those with higher HA contents. The elongation at the break of HA/PVP blend films displayed a pronounced increase compared to HA films. Moreover, infrared analysis confirmed the existence of interactions between HA and PVP. The blending of HA with PVP generated films with elasticity and better properties than homopolymer films.

## 1. Introduction

Viscometric and rheological measurements are commonly employed for the characterisation of polymer fluids [1,2,3,4,5,6,7]. Viscosity behaviour and flow properties are considered as rates of product quality for calculation in many processes involving fluid flow e.g., extraction, extrusion and filtration [8,9,10]. Based on the aforementioned investigations, one can design new materials with unique properties for specific applications, for example, wound healing or tissue engineering purposes. Additionally, the viscometric and rheological behaviours of polymers and their blends or composites provide valuable information regarding their viscosity, stability at various conditions and miscibility in blends with other compounds. Miscibility of polymers in solution is a vital indicator for the physical and surface properties of a blend. Additionally, it is a significant aspect for the production of new functional materials based on the mixing of biopolymers with other components. Nowadays, the use of biopolymers such as polysaccharides and proteins, obtained from renewable resources, are more favourable [2,4,9,11]. Hyaluronic acid (HA) is a natural, polyanionic polysaccharide produced from natural sources, including strains of *Streptococcus* bacteria, rooster combs, marine animals, etc. It is a linear polymer consisting of repeating disaccharide units of d-glucuronic acid and *N*-acetyl-d-glucosamine residues. HA and materials based on hyaluronan are widely used for many medical, pharmaceutical and cosmetic applications [11,12,13]. Additionally, it can be applied in different forms, such as solution, gel, thin films and sponge. HA is often mixed with other hydrophilic polymers to overcome the drawbacks of rapid degradation and poor mechanical properties [14,15,16,17,18]. The mixing of HA with other polymers allows the formation of new materials in which intermolecular interactions between different components can occur. In this research, we use poly(*N*-vinylpyrrolidone) (PVP) to modify HA materials for potential cosmetic, biomedical and packaging applications. It is well known that PVP is a synthetic, water-soluble, non-ionic polymer, which possesses many excellent properties such as film formation ability, biocompatibility, non-toxicity, remarkable chemical and thermal stability, excellent solubility in water and many organic solvents and applicability in the preparation of biomaterials [19,20,21,22,23]. In addition, PVP has been approved by the US Food and Drug Administration as a safe polymer for biological experiments; most that are tested are from the biomedical and pharmaceutical fields [21,24,25]. To the best of our knowledge, the miscibility, rheological and mechanical properties of HA blends with PVP have yet to be reported.

The aim of this work was to estimate the miscibility, rheological and mechanical properties of two HAs of various molecular weights, blended with PVP, on the basis of the viscometric technique, steady rheological measurements, mechanical tests and infrared analysis. Techniques used in this work allowed the study of homogeneity, miscibility, flow properties and intermolecular interactions. In addition, for cosmetic, food and pharmaceutical applications, it is important to determine the rheological properties at various temperatures in order to develop suitable compositions and their suitability under various conditions.

## 2. Materials and Methods

### 2.1. Materials

HA samples were purchased from Sigma-Aldrich (Poznan, Poland) and had a viscosity average molecular weight of 543 kg/mol for HA I and 1110 kg/mol for HA II. PVP had a viscosity average molecular weight of 818 kg/mol and was purchased from Sigma-Aldrich (Poznan, Poland). Viscosity average molecular weight was determined by the intrinsic viscosity in the solution using the Mark–Houwink–Sakurada equation [26]. The Mark–Houwink constants for HA were obtained from the literature [27] with values of K = 3.36 × 10^−2^ cm^3^/g and a = 0.79 at 25 °C in 0.1 mol/L NaCl. PVP in water had the constants of K = 3.93 × 10^−2^ cm^3^/g and a = 0.59 at 30 °C [28]. All reagents, materials and chemicals used in this study were purchased from POCh (Avantor, Gliwice, Poland) and Chempur (Piekary Slaskie, Poland). All materials were of analytical grade and used without any further purification.

Each polymer sample was separately dissolved in water at room temperature. HA/PVP blends were obtained by mixing aqueous solutions of HA and PVP in various weight ratios. HA weight fractions in the blends were 0, 0.2, 0.5, 0.8 and 1.

### 2.2. Fims Prepartion

The thin films were prepared by the solution casting method. In the first step, 2% (*m*/*v*) HA and 5% (*m*/*v*) PVP solutions were used to prepare blended solutions with different HA weight fractions (0, 0.2, 0.5, 0.8 and 1). In the second step, the polymer blend solutions were placed into plastic Petri dishes and dried at room temperature for 72 h. The films were peeled off and examined. All films were visually homogeneous, irrespective of composition.

### 2.3. Viscometric Technique

The viscometric studies were performed on dilute solutions of polymers (c < 0.5% (*m*/*v*)). The stock solutions of each blend were prepared and diluted, producing the five lower concentrations made through addition of the appropriate amount of water to the stock solutions. The reduced viscosity of pure polymer and HA/PVP blend solutions was determined using a Ubbelohde viscometer at 25 ± 0.1 °C immersed in a constant temperature bath. The flow time of solution was taken as the average of three readings with an accuracy of ±0.01 s. The viscometric data were calculated using the same methods described in previous reports [26,29].

### 2.4. Steady Shear Rheological Studies

Steady shear rheological measurements of solutions were performed on a Bohlin Visco BV 88 rotary viscometer with a concentric cylinder (Marlvern, Panalytical, Malvern, UK) at various temperatures (25–40 °C) and shear rates (20–1230 s^−1^). For this measurement, solutions of 2% (*m*/*v*) HA and 5% PVP (*m*/*v*) were prepared in distilled water. Rheological parameters (Ostwald–de Waele equation) were determined using the same methods described in previous papers [9,29].

### 2.5. Mechanical Tests

Mechanical properties were measured with a Z05 Zwick & Roell (Zwick&Roell, Ulm, Germany) at a crosshead speed of 50 mm/min in accordance with the standard procedure under dry conditions at room temperature [30]. All films were cut using the same shaper. The size of the test samples was 10 mm in width and 25 mm in parallel length. For each type of film, a minimum of five samples were tested. Tensile strength (TS), Young’s modulus (YM) and percentage elongation at the break (EB) were measured.

### 2.6. Infrared Spectroscopy (ATR-FTIR)

Spectra of the thin films were recorded using a Nicolet iS10 FTIR spectrophotometer (Thermo Fisher Scientific Inc., Waltham, MA, USA) in attenuated total reflectance (ATR) mode with a diamond crystal in the range of 4000–600 cm^−1^ during 64 scans, and at a resolution of 2 cm^−1^.

## 3. Results and Discussion

### 3.1. Viscometric Studies

The viscometric technique was conducted in order to determine the miscibility and molecular interactions of HA and PVP. In our previous reports [26,29], the main purpose of utilising the viscometric technique was to study interactions and miscibility in dilute polymer solutions. Briefly, the quantitative evaluation of miscibility by the viscometric technique was obtained by calculating miscibility parameters ($\Delta b_{m}$) as well as intrinsic viscosities, both theoretically (by the method described by Garcia et al. [31]) and experimentally, which were then plotted against the polymer solution concentrations. Miscibility parameter $\Delta b_{m}$ was calculated using Equation (1):

(1) $$\Delta b_{m}=b_{m}^{exp}-b_{m}^{id}$$

where $b_{m}^{exp}$ is the experimental interaction parameter (dL/g)^2^ and $b_{m}^{id}$ is the ideal interaction parameter (dL/g)^2^.

The experimental interaction parameter was obtained from the graph of reduced viscosity vs. polymer concentration using the classical Huggins equation, shown in Equation (2):

(2) $$\frac{{\left(\eta_{sp}\right)}_{m}}{c_{m}}={\left[\eta \right]}_{m}^{exp}+b_{m}^{exp}c_{m}$$

where $\frac{{\left(\eta_{sp}\right)}_{m}}{c_{m}}$ is the reduced viscosity (dL/g), c_m_ is the total concentration of the polymer blend (g/dL) and ${\left[\eta \right]}_{m}^{exp}$ is the experimental intrinsic viscosity (dL/g). The graph of $\frac{{\left(\eta_{sp}\right)}_{m}}{c_{m}}$ vs. $c_{m}$ provided a straight line, where the intercept and slope are respectively equal to ${\left[\eta \right]}_{m}^{exp}$ and $b_{m}^{exp}$. The ideal interaction parameter was expressed as Equation (3):

(3) $$b_{m}^{id}=w_{1}b_{1}^{2}+w_{2}b_{2}^{2}$$

where $w_{1}$ and $w_{2}$ are the weight fractions of polymer 1 and 2, respectively, and $b_{1}$ and $b_{2}$ are the interaction parameters of each homopolymer. In addition, Garcia et al. [31] proposed an additional criterion based on the difference between the experimental intrinsic viscosity (${\left[\eta \right]}_{m}^{exp}$) and the ideal intrinsic viscosity (${\left[\eta \right]}_{m}^{id}$). The ideal value was obtained from Equation (4):

(4) $${\left[\eta \right]}_{m}^{id}={\left[\eta \right]}_{1}w_{1}+w_{2}{\left[\eta \right]}_{2}$$

where ${\left[\eta \right]}_{1}$ and ${\left[\eta \right]}_{2}$ are the intrinsic viscosities of polymer 1 and 2, respectively.

**Figure 1:**
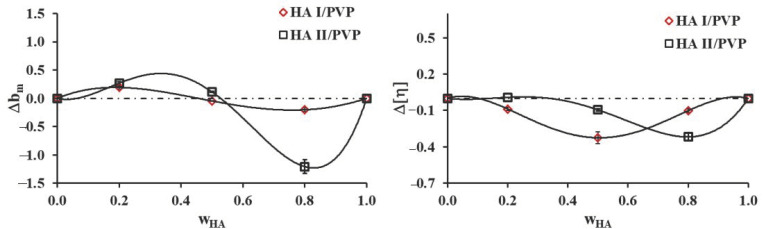
Values of miscibility parameters ($\Delta b_{m}$ and Δ[η] ) vs. HA weight fractions (w_HA_) in HA I/PVP and HA II/PVP blends.

The application of the method proposed by Garcia et al. [31] allowed the calculation of the miscibility parameters $\Delta b_{m}$ and Δ[η] to evaluate the degrees of miscibility in HA I/PVP and HA II/PVP blends. Values of $\Delta b_{m}$ > 0 and Δ[η] < 0 relate to the miscibility of polymer blend solutions, and values of $\Delta b_{m}$ < 0 and Δ[η] > 0 relate to the immiscibility. Moreover, values of $\Delta b_{m}$ > 0 imply the presence of attractive molecular interactions, while $\Delta b_{m}$ < 0 indicates repulsive molecular interactions. The Huggins graphs of reduced viscosity vs. polymer concentration (graphs not shown) showed a linear relationship for all polymer solutions over the entire composition range, indicating that the intrinsic viscosity values could be evaluated by linear extrapolation to zero concentration. Figure 1 presents miscibility parameters ($\Delta b_{m}$ and Δ[η]) vs. HA weight fractions (w_HA_) in HA I/PVP and HA II/PVP blends.

As shown in Figure 1, $\Delta b_{m}$ values were positive, with the exception of the blend with high HA content (w_HA_ > 0.5). In the case of HA II/PVP blends, the negative value of $\Delta b_{m}$ was approximately six times bigger than that of HA I/PVP blends. The observed changes in the miscibility of HA and PVP were related to the molecular weights of the HAs. It is well known that polymers with relatively similar low molecular weights tend to be more miscible [32]. The higher molecular weight of HA II was probably responsible for the clearly negative value of the miscibility parameter. Thus, HA II/PVP blends had poor miscibility in solution compared to HA I/PVP blends. According to Δ[η] values presented in Figure 1, HA/PVP blends were miscible due to the experimental intrinsic viscosities being smaller than the ideal intrinsic viscosity values, indicating negative deviations. In certain circumstances, the molecular interactions between chains hinder interactions between the polymer chain and solvent, weakening the solvation impact and leading to reduced viscosity [29,33]. Hence, the negative deviations were observed.

### 3.2. Steady Shear Rheological Studies

Steady shear measurements were carried out to evaluate the rheological properties of HA, PVP and their blends at different temperatures of 25 °C, 30 °C, 35 °C and 40 °C.

**Figure 2:**
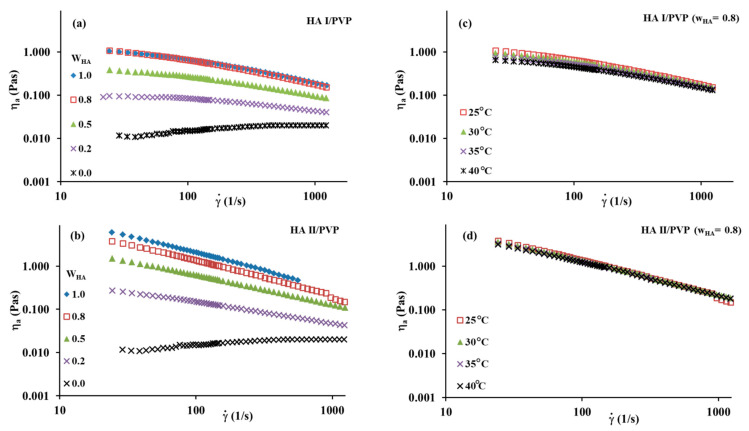
(**a**,**b**) Plots of apparent shear viscosity $\eta_{a}$ vs. shear rate $\dot{\gamma }$ of HA and PVP and HA/PVP blends at 25 °C; (**c**,**d**) plots of apparent shear viscosity $\eta_{a}$ vs. shear rate $\dot{\gamma }$ of a HA/PVP (w_HA_ = 0.8) blend for temperatures ranging from 25 °C to 40 °C.

Figure 2 shows the apparent shear viscosity as a function of shear rate for homopolymers as well as composition blends. In the case of the pure HA solution with a concentration of 2% and the HA/PVP blend solutions, a shear-thinning behaviour was observed that indicated a strong shear dependence of the apparent viscosity. This typical shear-thinning behaviour (pseudoplastic nature) was associated with the disentanglement of polymer molecules under increasing shear forces and the orientation of molecules along the streamline of the flow [4,9,29]. Thus, we observed a decrease in apparent viscosity with an increase in shear rate. The viscosity plots of all HA/PVP blends were between the homopolymer plots. In the case of the pure PVP solution with a concentration of 5%, the apparent viscosity of the solution increased with the increasing shear rate, indicating a shear-thickening behaviour. This behaviour was due to molecular conformational changes induced by flow as well as association of polymer molecules. Figure 2c and d present the viscosity plots vs. shear rate of a HA/PVP (w_HA_ = 0.8) blend at different temperatures. The HA/PVP blend exhibited a weak temperature effect towards the apparent viscosity (decreased slightly) in the temperature range between 25 °C and 40 °C. Similar changes were also observed for other blended solutions (curves not shown).

The Ostwald–de Waele model was applied to determine the fluid flow properties of the polymer blend solutions as a function of HA content [4,9,29]. The Ostwald–de Waele equation is expressed in Equation (5):

(5) $$\eta_{a}=k{\dot{\gamma }}^{n-1}$$

where *k* and *n* are rheological parameters, *k* is the consistency index (Pas)*^n^* and *n* is the non-Newtonian index (dimensionless). The presented rheological parameters show the impact of HA content in the blend on the non-Newtonian flow of the polymer blend solution. For non-Newtonian fluids, the value of *n* was not equal to 1. A value of *n* < 1 indicates shear-thinning behaviour, whereas a value of *n* > 1 suggests shear-thickening behaviour.

The shear-thinning and shear-thickening behaviours of all polymer solutions and their blends were well characterised by the Ostwald–de Waele model, which provided good adjustment of the experimental data. n and k parameters calculated by the Ostwald–de Waele equation are listed in Table 1.

The non-Newtonian indices (*n*) of HA and HA/PVP blend solutions were below 1 in the temperature range of 25 °C–40 °C, indicating a shear-thinning effect. As the molecular weight of HA increased to 1110 kg/mol, a pronounced reduction in the value of *n* from 0.47 to 0.16 at 25 °C was observed. The low n value was related to the entanglement of HA II molecules and those with a high degree of association in the solution. A temperature increase of 10 °C only slightly impacted the value of n (e.g., increase from 0.16 to 0.17 in HA II solution). In the case of PVP solutions, where a shear-thickening effect occurred, the value of n was above 1. k parameter values increased with HA contents in the blend solutions but decreased with increasing temperature.

### 3.3. Mechanical Tests

**Figure 3:**
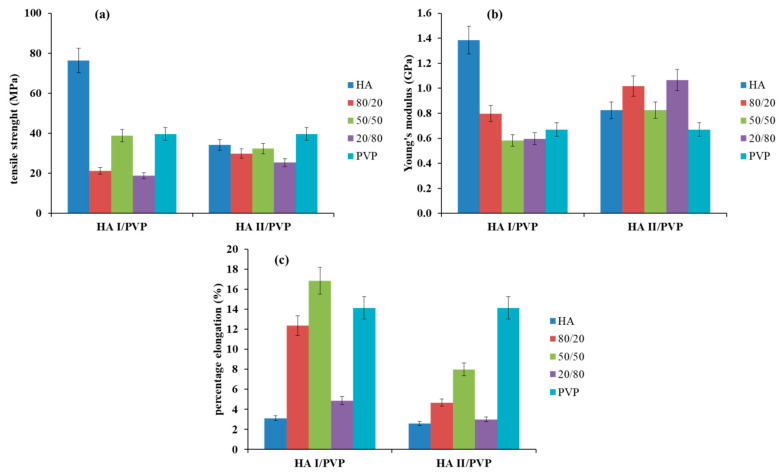
Mechanical properties of HA, PVP and HA/PVP blends: (**a**) tensile strength (TS), (**b**) Young’s modulus (YM) and (**c**) percentage elongation at the break (EB). w_HA_—HA weight fraction.

Tensile tests were conducted to evaluate the mechanical properties such as tensile strength (TS), Young’s modulus (YM) and percentage elongation at the break (EB) for HA, PVP and HA/PVP blend films. As shown in Figure 3, the values of TS and YM were similar for PVP and HA II films but smaller for HA I films.

HA I/PVP blend films exhibited lower TS and YM values compared to HA I films. In the case of HA II/PVP blend films with w_HA_ ≥ 0.5, TS values did not change in the blends and were similar to those for pure HA II films within experimental error. HA II/PVP blend films with w_HA_ = 0.2 had reduced TS values but exhibited higher YM values compared to PVP films. EB values were the highest for PVP films. Therefore, after the addition of PVP to HA solutions, all films showed increased EB values. The maximum EB value was achieved by HA I/PVP films with w_HA_ = 0.5. Thus, HA blends with PVP allow for the preparation of films with improved elastic properties. This was due to interactions between polymer molecules. The addition of PVP to HA solutions promoted reduced intramolecular hydrogen bonds between HA chains and created new hydrogen bonds between the polymer molecules. Thus, interactions change the polymer networks, leading to improved selected properties of the polymer films.

### 3.4. Infrared Spectroscopy

**Figure 4:**
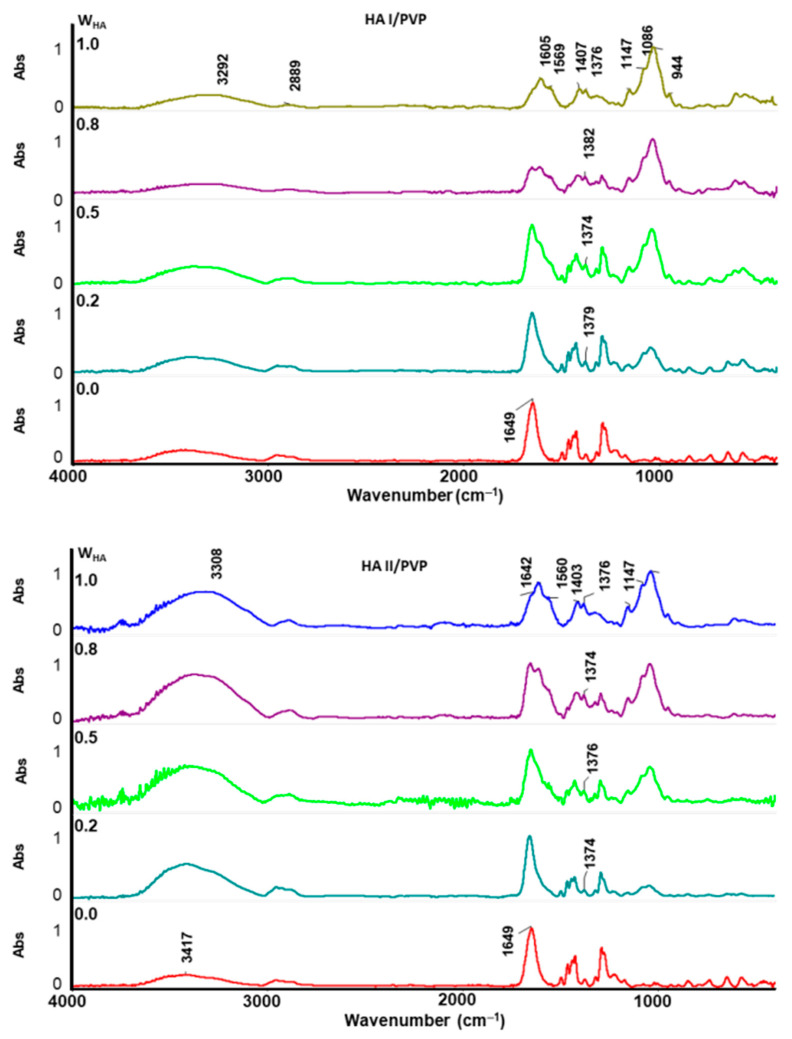
ATR infrared spectra of HA, PVP and HA/PVP blend films. w_HA_—HA weight fraction.

Infrared spectroscopy was carried out to investigate the intermolecular interactions between polymers (Figure 4).

The spectra of HA films exhibited all typical bands and peaks characteristic of polysaccharides. The strong bands between 900 and 1200 cm^−1^ relate to the vibrations of C-O, C-O-C glycosidic and C-O-H bonds. The stretching vibrations of hydroxyl groups (OH) appeared at approximately 3300 cm^−1^ in HA films’ spectra, which overlapped with the NH stretching bands. The carbonyl stretching vibrations of the carboxylate group of HA films appeared at 1605 cm^−1^ and 1403 cm^−1^ [15,34,35]. In the case of PVP films, the spectra show intense bands at 1649 cm^−1^, corresponding to the stretching vibrations of the carbonyl group (C=O) in the pyrrolidone ring [23,36]. Analyses of HA/PVP blend spectra suggested interactions existing between polymer molecules, which were associated with the OH and carboxylate groups in HA and the carbonyl group (C=O) in PVP. As shown in Figure 4, the intensity of the carboxylate bands at 1605 cm^−1^ and 1403 cm^−1^ and hydroxyl bands at 3310 cm^−1^ were reduced, which indicated that some of the carboxylic and hydroxyl groups generated new hydrogen bonds. Additionally, the sharp peaks at approximately 1380 cm^−1^ in all HA/PVP spectra were attributed to carboxyl groups (COO^−^) that take part in one or more hydrogen bonds [34,37]. Furthermore, broadening and shifting of the carbonyl bands from 1649 cm^−1^ to lower frequencies confirmed the interactions between HA and PVP [23]. Furthermore, the absorption bands between 3300 cm^−1^ and 3500 cm^−1^ were assigned to the intra- and intermolecular hydrogen bonding of OH groups [34,38]. Thus, the shifts of this region indicated that C=O groups of PVP were involved in hydrogen bond formation with OH groups of HA at the expense of the hydrogen bonding interaction in HA. The infrared spectroscopy confirmed the intermolecular interactions between polymer chains that impact the viscosity behaviour and mechanical properties.

## 4. Conclusions

In this study, the miscibility as well as rheological and mechanical properties of two HAs possessing various molecular weights, blended with PVP, were examined. The viscometric technique, steady shear measurements, infrared spectroscopy and mechanical results clearly highlighted intermolecular interactions between HA and PVP. HA blends with PVP were miscible, with the exception of the blend with high HA content (w_HA_ > 0.5). Solutions of HA and HA/PVP blends exhibited shear-thinning behaviour. Analysis revealed that temperature had little effect on HA/PVP blend solutions’ apparent viscosity. In addition, the rheological data were well simulated by the Ostwald–de Waele model. Parameters *n* of all HA/PVP blends were below one, indicating pseudoplasticity, which increased with increasing HA contents in the blends. TS and EB values were higher for HA/PVP films with w_HA_ = 0.5 compared to films with higher HA contents. EB values for HA/PVP blend films showed pronounced increases compared to HA films. The prepared HA/PVP films were determined as elastic due to intermolecular interactions via hydrogen bonding enhancing the properties of the materials. Infrared spectroscopy examined the relative changes in OH, COOH and C=O intensities, which showed that new hydrogen bonds were formed between molecules. Thus, HA/PVP blends may be utilised for biomedical applications, such as wound healing and tissue engineering, cosmetics, e.g., hair care, and packaging.

## Figures and Tables

**Table 1:** Parameters *n* and *k* obtained by the Ostwald–de Waele equation from steady shear measurements.

w_HA_	25 °C			35 °C		
	*n*	*k* (Pas)*^n^*	R^2^	*n*	*k* (Pas)*^n^*	R^2^
HA I/PVP						
1.0	0.47	7.78	0.993	0.44	9.99	0.993
0.8	0.44	8.53	0.994	0.50	5.34	0.994
0.5	0.56	2.04	0.997	0.62	1.21	0.996
0.2	0.72	3.18	0.999	0.77	0.19	0.998
0.0	1.21	5.6 × 10^−3^	0.999	1.30	2.0 × 10^−3^	0.998
HA II/PVP						
1.0	0.16	101.4	0.965	0.17	90.1	0.975
0.8	0.20	55.2	0.995	0.22	45.3	0.994
0.5	0.31	14.8	1.00	0.33	11.5	0.999
0.2	0.50	1.54	1.00	0.52	1.15	1.00

w_HA_—HA weight fraction.

## Data Availability

Data is contained within the article.

## Sample Availability

Samples of the compounds are not available from the authors.

## References

[1] Ghannan M.T., Esmail M.N. (1997). Rheological properties of carboxymethyl cellulose. J. Appl. Polym. Sci..

[2] Cheng Y., Brown K.M., Prud’Humme R.K. (2002). Characterization and intermolecular interactions of hydroxypropyl guar solutions. Biomacromolecules.

[3] Jukić A., Rogošić M., Bolarić I., Tomašek L., Janović Z. (2004). Viscometric study of miscibility and interactions of some polyolefines and poly(alkyl methacrylates) in dilute xylene solutions. J. Mol. Liq..

[4] Wang S., He L., Guo J., Zhao J., Tang H. (2015). Intrinsic viscosity and rheological properties of natural and substituted guar gum in seawater. Int. J. Biol. Macromol..

[5] Brunchi C.E., Bercea M., Morariu S., Avadanei M. (2016). Investigations on the interactions between xanthan gum and poly(vinyl alcohol) in solid state and aqueous solutions. Eur. Polym. J..

[6] Yang H., Duan L., Li Q., Tian Z., Li G. (2018). Experimental and modeling investigation on the rheological behavior of collagen solution as a function of acetic concentration. J. Mech. Behav. Biomed. Mater..

[7] Wang X., Zhou D., Zhu G., Guo C. (2021). Rheological properties of two high polymers suspended in ab abrasive slurry jet. e-Polymers.

[8] Ma J., Lin Y., Chen X., Zhao B., Zhang J. (2014). Flow behavior, thixotropy and dynamic viscoelasticity of sodium alginate aqueous solution. Food Hydrocoll..

[9] Tian Z., Duan L., Wu L., Shen L., Li G. (2016). Rheological properties of glutaraldehyde-crosslinked collagen solutions analyzed quantitatively using mechanical models. Mater. Sci. Eng. C.

[10] Ahmad H.M., Kamal M.S., Al-Harthi M.A. (2018). Rheological and filtration properties of clay-polymer systems: Impact of polymer structure. Appl. Clay Sci..

[11] Jummaat F., Yahya E.B., Khalil A., Adnan A.S., Alqadhi A.M., Abdullah C.K., Atty Sofea A.K., Olaiya N.G., Abdat M. (2021). The role of biopolymer-based materials in obstetrics and gynecology applications: A review. Polymers.

[12] Hinchliffe J.D., Madappura A.P., Mohamed S.M.D.S., Roy I. (2021). Review: Biomedical applications of bacteria-derived polymers. Polymers.

[13] Valachová K., Šoltěs L. (2021). Versatile use of chitosan and hyaluronan in medicine. Molecules.

[14] Croll T.I., O’Connor A.J., Stevens G.W., Cooper-White J.J. (2006). A Blank Slate? Layer-by layer deposition of hyaluronic acid and chitosan onto various surface. Biomacromolecules.

[15] Lopes T.D., Riegel-Vidotti L.C., Grein A., Tischer C.A., de Sousa Faria-Tischer P.C. (2014). Bacterial cellulose and hyaluronic acid hybrid membranes: Production and characterization. Int. J. Biol. Macromol..

[16] Pan N.C., Bersaneti G.T., Mali S., Colabone Celligoi M.A.P. (2020). Films based on blends polyvinyl alcohol and microbial hyaluronic acid. Braz. Arch. Biol. Technolo..

[17] Sionkowska A., Gadomska M., Musiał K., Piątek J. (2020). Hyaluronic acid as a component of natural polymer blends for biomedical applications: A review. Molecules.

[18] Chun-Gamboa M.G., Cámara Perer C.M., Aguilar Ayala F.J., Vargas-Coronado R.F., Cauich-Rodriguez J.V., Escobar-Garcia D.M., Sánchez- Vargas L.O., Pacheo N., San Román del Barrio J. (2021). Antibacterial behavior of chitosan-sodium hyaluronate-PEGDE crosslinked films. Appl. Sci..

[19] Lim J.L., Kang M.L., Lee W.K. (2014). Lotus-leaf-like structured chitosan-polyvinyl pyrrolidone films as an anti-adhesion barrier. Appl. Surf. Sci..

[20] Hasan A., Waibhaw G., Tiwari S., Dharmalingam K., Shukla I., Pandey L.M. (2017). Fabrication and characterization of chitosan, polyvinylpyrrolidone, and cellulose nanowhiskers nanocomposite films for wound healing drug delivery applications. J. Biomed. Mater. Res. Part A.

[21] Teodorescu M., Bercea M., Morariu S. (2019). Biomaterials of PVA and PVP in medical and pharmaceutical applications. Perspective and challenges. Biotechnol. Adv..

[22] Lobo B., Veena L. (2021). Experimental investigations on nano-titania incorporated polyvinyl alcohol-polyvinyl pyrrolidone composite films. Polym.-Plast. Technol. Mater..

[23] Kumar R., Mishara I., Kumar G. Synthesis and evaluation of mechanical property of chitosan/PVP blend through nanoindentation-a nanoscale study. J. Polym. Environ..

[24] D’Souza A.J.M., Schowen R.L., Topp E.M. (2004). Polyvinylpyrrolidone-drug conjugate: Synthesis and release mechanism. J. Control. Release.

[25] Smith L.E., Rimmer S., MacNeil S. (2006). Examination of the effect of poly(N-vinylpyrrolidone) hydrogel in direct and indirect contact with cells. Biomaterials.

[26] Lewandowska K. (2005). The miscibility of poly(vinyl alcohol)/poly(N-vinylpyrrolidone) blends investigated in dilute solutions and solids. Eur. Polym. J..

[27] Garcia-Abuin A., Gomez-Diaz D., Navaza J.M., Regueiro L., Vidal-Tato I. (2011). Viscosimetric behaviour of hyaluronic acid in different aqueous solutions. Carbohydr. Polym..

[28] Cerny L.C., Helminiak T.E., Meier J.F. (1960). Osmotic pressures of aqueous polyvinylpyrrolidone solutios. J. Polym. Sci..

[29] Lewandowska K. (2020). Miscibility studies of hyaluronic acid and poly(vinyl alcohol) blends in various solvents. Materials.

[30] Polish Norm PN-81/C-89034 (ISO 527-1 and 527-2) (2012). International Standard: Plastic—Determination of Tensile Properties. https://www.iso.org/standard/56045.html.

[31] Garcia R., Melad O., Gómez C.M., Figueruelo J.E., Campos A. (1999). Viscometric study on the compatibility of polymer-polymer mixtures in solution. Eur. Polym. J..

[32] Dondos A., Christopoulou V., Papanagopoulos D. (1999). The influence of the molecular mass of two incompatible polymers on their miscibility in the solid state without compatibilizer after casting from solution. J. Polym. Sci. Part B Polym. Phys..

[33] Pingping Z., Haiyang Y., Shiqiang W. (1998). Viscosity behavior of poly-ε-caprolactone (PCL)/poly(vinyl chloride) (PVC) blends in various solvents. Eur. J. Polym..

[34] Haxaire K., Marėchal Y., Milas M., Rinaudo M. (2003). Hydration of polysaccharide hyaluronan observed by IR spectrometry. I. Preliminary experiments and band assignments. Biopolymers.

[35] Cai Z., Zhang F., Wei Y., Zhang H. (2017). Freeze-thaw-induced gelation of hyaluronan: Physical cryostructuration correlated with intermolecular associations and molecular conformation. Macromolecules.

[36] Lewandowska K. (2011). Miscibility and interactions in chitosan acetate/poly(N-vinylpyrrolidone) blends. Thermochim. Acta.

[37] Lee E.J., Kang E.S., Kang W., Huh K.M. (2020). Thermo-irreversible glycol chitosan/hyaluronic acid blend hydrogel for injectable tissue engineering. Carbohydr. Polym..

[38] Wang B., Wang J., Li D., Ren K., Ji J. (2012). Chitosan/poly(vinyl pyrollidone) coatings improve the antibacterial properties of poly(ethylene terephthalate). Appl. Surf. Sci..

